# Supplementary material for: Comparison of optimal bowel cleansing effects of 1L polyethylene glycol with ascorbic acid versus sodium picosulfate with magnesium citrate: A randomized controlled study
Source: PLoS One. 2022 Dec 30;17(12):e0279631. doi: 10.1371/journal.pone.0279631 (PMC9803231; doi:10.1371/journal.pone.0279631)
Supplement: S1 File — (PDF) [file pone.0279631.s002.pdf]

1. How was the taste of the bowel cleansing agent for this colonoscopy? (Please check √ in the relevant □ box)

0	1	2	3	4	5	6	7	8	9	10
□	□	□	□	□	□	□	□	□	□	□

Very disliked ←→ Very liked

2. How do you feel about taking the bowel cleansing agent for this colonoscopy? (Please check √ in the relevant □ box)

0	1	2	3	4	5	6	7	8	9	10
□	□	□	□	□	□	□	□	□	□	□

Very disliked ←→ Very liked

3. How easy was it to take the bowel cleansing agent for this colonoscopy? (Please check √ in the relevant □ box)

0	1	2	3	4	5	6	7	8	9	10
□	□	□	□	□	□	□	□	□	□	□

Very difficult ←→ Very easy

4. How is the overall satisfaction of the bowel cleansing agent you have taken this colonoscopy? (Please check √ in the relevant □ box)

0	1	2	3	4	5	6	7	8	9	10
□	□	□	□	□	□	□	□	□	□	□

Very dissatisfied ←→ Very satisfied

5. How much did you take bowel cleansing agent for this colonoscopy (Please check √ in the relevant □ box)

□ The total dose was taken.

□ Failed to take full dose.

6. Would you accept taking the same bowel cleansing agent for your next colonoscopy? (Please check √ in the relevant □ box)

- □ Yes I accept
- □ No I do not accept

7. Did you experience any of the following symptoms while taking the bowel cleansing agent for this colonoscopy? (Please check √ in the relevant □ box.)

**Symptoms**	**Occurrence**	
Nausea	□ Yes	□ No
Vomiting	□ Yes	□ No
Thirstiness	□ Yes	□ No
Abdominal pain	□ Yes	□ No
Abdominal distension	□ Yes	□ No
Fecal incontinence	□ Yes	□ No
General weakness	□ Yes	□ No
Tingling sensation	□ Yes	□ No
Insomnia	□ Yes	□ No
Mental change	□ Yes	□ No
Seizure	□ Yes	□ No
Dizzines	□ Yes	□ No
