# Supplementary material for: Comparison of optimal bowel cleansing effects of 1L polyethylene glycol with ascorbic acid versus sodium picosulfate with magnesium citrate: A randomized controlled study
Source: PLoS One. 2022 Dec 30;17(12):e0279631. doi: 10.1371/journal.pone.0279631 (PMC9803231; doi:10.1371/journal.pone.0279631)
Supplement: S2 File — (ZIP) [file pone.0279631.s003.zip › Study protcol, statement, consent form, consort check list/Consent form for clinical trial subject(English).docx]

**Consent form for clinical trial subject**

Agreement
Before signing the consent form, please check the following items once again and mark them directly in the appropriate box.

|  | **“Yes”** |
| --- | --- |
| 1. I have read the description of this study and have fully discussed it with the researcher in charge | □ |
| 2. I have heard about the risks (disadvantages) and benefits (benefits) that may arise from participating in this study, and what to do in case of side effects, and I have received satisfactory answers to my questions.. | □ |
| 3. I understand that the researcher must immediately report to me if new information about me is collected in the course of the research process, or if any disadvantages other than the risks described at the time of consent arise. | □ |
| 4. I voluntarily consent to participate in this study, and I have not been subjected to any coercion or undue influence. | □ |
| 5. I have been informed about the type and scope of information about myself (research subject) to be collected in this study, and it is sufficient that the researcher collects and processes it within the scope permitted by current laws and institutional bioethics committee regulations. I understand. | □ |
| 6. I will keep my personally identifiable information kept confidential when the researcher or an authorized representative conducts research or manages the results, and when the health authorities, school authorities, and the Korea Institute of Health and Medical Research Institutional Bioethics Committee conduct a fact-finding investigation I agree to direct viewing. | □ |
| 7. I understand that I may withdraw from this study at any time and that this decision will not disadvantage me in any way. | □ |
| 8. My signature indicates that I have received a copy of this consent form, and I will keep a copy until the end of my participation in the study. And the name, signature, and date were all handwritten. | □ |
| 9. (In the case of a representative) The research subject was unable to understand this study and could not consent by hand, so the representative fully understood and signed on his/her behalf. | □ |
| 10. (In the case of a fair witness) The research subject and his/her representative participated in the entire consent process because they could not read the consent form, research subject description, and other documented information confirmed that. | □ |

| Name of study subject |  | signature |  | signature date |  |
| --- | --- | --- | --- | --- | --- |
| Representative's name (if necessary) |  | signature |  | signature date |  |
| Reasons for needing an representative |  | | Relationship withdlqgh study subjects | |  |
| Name of fair witness(if, need) |  | signature |  | signature date |  |
| Reasons for needing fair witness |  | | | | |
| ● Representative means legal representative. | | | | | |
| 1. Representatives in research subject to the Pharmaceutical Affairs Act/Medical Device Act  : A test subject's representative means a person who is the parent, spouse, or guardian of a research subject and can make a decision on whether or not to participate in a clinical trial on behalf of the subject | | | | | |
| 2. Representatives in research subject to the Bioethics Act  2.1. legally authorized representative  A legal representative refers to a person whose power of representation takes effect directly under the provisions of the Act without being delegated.  2.2. If there is no legal representative, the spouse, lineal ascendant, lineal descendant shall be in the order of succession, but if there are several lineal ascendants or lineal descendants, the decision is made by agreement.  * In the case of research involving minors, for research that requires the consent of two parents, the legal representative is added and used  ------------------------------------------------------------ --------------------  < Below the dotted line is the field for the principal investigator (physician in charge). >  The principal investigator explained the purpose, method, patient's rights, confidentiality, etc. of the research study in detail to the above patient, and confirms that the patient himself/herself decided to participate in this study and signed the agreement.  Year Month Day | | | | | |
|  | | | | | |
|  | | | | | |
| Responsible/Collaborative Researcher Name |  | signature |  | | |
