# Supplementary material for: Comparison of optimal bowel cleansing effects of 1L polyethylene glycol with ascorbic acid versus sodium picosulfate with magnesium citrate: A randomized controlled study
Source: PLoS One. 2022 Dec 30;17(12):e0279631. doi: 10.1371/journal.pone.0279631 (PMC9803231; doi:10.1371/journal.pone.0279631)
Supplement: S2 File — (ZIP) [file pone.0279631.s003.zip › Study protcol, statement, consent form, consort check list/Consent form for clinical trial subject(Korean).docx]

**[서식 11]**

**동 의 서**

* 동의서에 서명하시기 전에 다음 사항을 다시 한번 확인하시고 해당 칸에 직접 표기하여 주시기 바랍니다.

|  | **“예”** |
| --- | --- |
| 1. 나는 이 연구에 대한 설명문을 읽었으며 담당 연구원과 이에 대하여 충분히 의논하였습니다. | □ |
| 2. 나는 이 연구 참여로 인해 발생할 수 있는 위험(불이익)과 이득(혜택) 그리고 부작용 발생 시 조치사항에 대하여 들었으며 나의 질문에 만족할 만한 답변을 얻었습니다. | □ |
| 3. 나는 연구 진행 과정에서 나에 대한 새로운 정보를 수집하거나, 동의 당시 설명받은 위험 이외의 불이익이 발생한 경우 연구자가 나에게 즉시 보고해야 한다는 것을 알고 있습니다. | □ |
| 4. 나는 이 연구에 참여하는 것에 대하여 자발적으로 동의하며, 어떠한 강제나 부당한 영향을 받지 않았습니다. | □ |
| 5. 나는 이 연구에서 수집될 나(연구대상자)에 대한 정보의 종류와 범위에 대한 설명을 들었으며, 이는 현행 법률과 기관생명윤리위원회 규정이 허용하는 범위 내에서 연구자가 수집하고 처리한다는 것을 충분히 이해하였습니다. | □ |
| 6. 나는 담당 연구자나 위임 받은 대리인이 연구를 진행하거나 결과 관리를 하는 경우와 보건 당국, 학교 당국 및 한국보건의료연구원 기관생명윤리위원회가 실태 조사를 하는 경우에는 비밀로 유지되는 나의 개인 신상 정보를 직접적으로 열람하는 것에 동의합니다. | □ |
| 7. 나는 언제라도 이 연구의 참여를 철회할 수 있고 이러한 결정이 나에게 어떠한 불이익도 되지 않을 것이라는 것을 알고 있습니다. | □ |
| 8. 나의 서명은 이 동의서의 사본을 받았다는 것을 뜻하며 연구 참여가 끝날 때까지 사본을 보관하겠습니다. 그리고 성명, 서명 그리고 날짜 모두 자필로 기록하였습니다. | □ |
| 9. (대리인인 경우) 연구대상자는 이 연구에 대해 이해할 수 없는 상태이고 자필로 동의할 수 없어 대리인이 대신 충분히 이해하고 자필로 서명하였습니다. | □ |
| 10. (공정한 입회자인 경우) 연구대상자와 대리인이 동의서 서식, 연구대상자설명서 및 기타 문서화된 정보를 읽을 수 없어서 동의 전 과정에 참여하였으며 연구대상자(대리인)의 자유의사로 이 연구 참여에 동의하였다는 것을 확인하였습니다. | □ |

| 연구대상자 성명 |  | 서명 |  | | 서명일자 |  | |
| --- | --- | --- | --- | --- | --- | --- | --- |
| 대리인 성명(필요시) |  | 서명 |  | | 서명일자 |  | |
| 대리 상황 및 사유 |  | | | 연구 대상자와 관계 | | |  |
| 입회자 성명(필요시) |  | 서명 |  | | 서명일자 |  | |
| 입회 상황 및 사유 |  | | | | | | |
| - 대리인은 법적 대리인을 의미합니다.   1. 약사법/의료기기법 적용 대상 연구에서의 대리인  시험대상자의 대리인이란, 연구대상자의 친권자ㆍ배우자 또는 후견인으로서, 시험대상자를 대신하여 연구대상자의 임상시험 참여 유무에 대한 결정을 내릴 수 있는 사람을 말한다.  2. 생명윤리법 적용 대상 연구에서의 대리인  2.1. 법정대리인 (legally authorized representative)  법정대리인이란 위임을 받지 않고도 직접 법률의 규정에 의하여 대리권의 효력이 발생하는 자를 말한다.  2.2. 법정대리인이 없는 경우 배우자, 직계존속, 직계비속의 순으로 하되 직계존속 또는 직계비속이 여러 사람일 경우 협의하여 정하고, 협의가 되지 아니하면 연장자가 대리인이 된다.  * 미성년자의 대상 연구의 경우, 부 또는 모 2명의 동의가 필요한 연구에 대하여는 법정대리인 항목을 추가하여 사용 | | | | | | | |
| **----------------------------------------------------------------------** | | | | | | | |
| < 점선 아래는 연구책임자(담당 의사) 기재란입니다. > | | | | | | | |
| 연구책임자는 본 조사연구의 목적, 방법, 환자의 권리, 비밀보장 등에 대해서 상세히 상기 환자에게 설명해 주었고, 환자 스스로 이 연구에 참여할 것을 결정하고 서명하였음을 확인합니다. | | | | | | | |
| **년 월 일** | | | | | | | |
| 책임/공동연구자 성명 |  | 서명 |  | | | | |
