# Supplementary material for: Comparison of optimal bowel cleansing effects of 1L polyethylene glycol with ascorbic acid versus sodium picosulfate with magnesium citrate: A randomized controlled study
Source: PLoS One. 2022 Dec 30;17(12):e0279631. doi: 10.1371/journal.pone.0279631 (PMC9803231; doi:10.1371/journal.pone.0279631)
Supplement: S2 File — (ZIP) [file pone.0279631.s003.zip › Study protcol, statement, consent form, consort check list/Full-length protocol.(English).docx]

**PROJECT TITLE:**

**Randomized clinical trial on efficacy and safety of 1L polyethylene glycol with ascorbic acid versus sodium picosulfate with magnesium citrate as preparation for colonoscopy**

**Participating Institution-1**

NAME of INSTITUTION: College of medicine, Chosun University

**Investigator-1**

TITLE: Professor

NAME: Jun Lee

CONTACT E-MAIL: leejun@chosun.ac.kr

**Participating Institution-1**

NAME of INSTITUTION: College of medicine, Chosun University

**Investigator-2**

TITLE: Clinical Assistant Professor

NAME: Seong Jung Kim

CONTACT E-MAIL: ygegh@hanmail.net

**Participating Institution-2**

NAME of INSTITUTION: Jeonbuk National University Medical School

**Investigator-3**

TITLE: Professor

NAME: Sang-Wook Kim

CONTACT E-MAIL: clickm@jbnu.ac.kr

**Participating Institution-3**

NAME of INSTITUTION: Wonkwang University School of Medicine

**Investigator-4**

TITLE: Clinical Assistant Professor

NAME: Hyo-Yeop Song

CONTACT E-MAIL: bfsongc@hanmail.net

**Participating Institution-3**

NAME of INSTITUTION: Wonkwang University School of Medicine

**Investigator-5**

TITLE: Professor

NAME: Geom Seog Seo

CONTACT E-MAIL: medsgs@wku.ac.kr

**Participating Institution-4**

NAME of INSTITUTION: Chonnam National University Medical School

**Investigator-6**

TITLE: Clinical Assistant Professor

NAME: Dong-Hyun Kim

CONTACT E-MAIL: bono343@naver.com

**Participating Institution-4**

NAME of INSTITUTION: Chonnam National University Medical School

**Investigator-7**

TITLE: Assistant Professor

NAME: Dae-Seong Myung

CONTACT E-MAIL: myungdaeseong@daum.net

**Participating Institution-4**

NAME of INSTITUTION: Chonnam National University Medical School

**Investigator-8**

TITLE: Professor

NAME: Hyun-Soo Kim

CONTACT E-MAIL: dshskim@chonnam.ac.kr

**Participating Institution-4**

NAME of INSTITUTION: Chonnam National University Medical School

**Investigator-9**

TITLE: Professor

NAME: Young-Eun Joo

CONTACT E-MAIL: yejoo@chonnam.ac.kr

**Participating Institution-5**

NAME of INSTITUTION: Department of Preventive Medicine, College of medicine, Chosun University

**Investigator-10**

TITLE: Assistant Professor

NAME: So-Yeong Kim^5^

CONTACT E-MAIL: soyeong4897@chosun.ac.kr

**1. SUMMARY OF THE PROJECT**

**1.1 Background**

Various low-volume bowel cleansing formulations that improve compliance have been approved and are being used in clinical practice.

**1.2 Study aim**

The purpose of this study is to compare the effectiveness of 1 L polyethylene glycol (PEG) with ascorbic acid with that of sodium picosulfate (PICO) with magnesium citrate.

**1.3 Study design**

This is a multicenter, randomized controlled, non-inferiority study to confirm the effectiveness and safety of 1L PEG with ascorbic acid.

**2. INTRODUCTION**

The prevalence of colorectal cancer in Korea is 69.3 in men and 45.9 in women per 100,000 people, making it the third most common cancer.^1^ Since more than 80% of colorectal cancers progress to the adenoma-cancerous process, removal of adenomas through colonoscopy can significantly reduce the incidence and mortality of colorectal cancer.^2,3^ However, even though colonoscopy was performed appropriately, it has been reported that about 6-8% of interval cancers. ^3,4^ Bowel preparation is one of the most important indicators for quality of the colonoscopy. However, inadequate bowel preparation is reported in approximately a quarter of all colonoscopies, which may reduce the detection rate of adenomas, delay procedure time, and increase the risk of side effects of the procedure. ^5,6^ An ideal bowel preparation should have high efficacy, ensure stability, and have high medication compliance. Although the 4 L PEG split-dose regimen provides high quality bowel cleansing, its large volume results in poor satisfaction, tolerability, and compliance.^6,7^ Various low-dose (2 L) cleansing agents have been newly developed, and several studies have confirmed that they are not inferior to 4 L PEG.^8,9,10^ However, low-dose cleansing agents still have a large volume and bad taste, so there is no preparation that can satisfy all patients so far. 1L PEG plus ascorbic acid, an ultra-low-dose laxative, was compared to the existing 2L PEG plus ascorbate through a phase 3 study, and similar results were confirmed in terms of the effect of bowel preparation, the patient's preference for repeating, and side effects. However, comparative studies between 1L PEG plus ascorbic acid and other low-dose laxatives are lacking so far.

**3. STUDY OBJECTIVES**

**3.1 Hypothesis**

1L PEG plus ascorbic acid and other low-dose laxatives is not inferior to other low-dose laxatives in efficacy, safety, and compliance.

**3.2 Overall aim**

The purpose of this study is to evaluate the efficacy, safety, compliance, and satisfaction of bowel preparation formulation by comparing the efficacy of 1 L PEG with ascorbic acid (Cleanviewal powder, Taejoon Pharm. Co, Seoul Korea) with that of sodium picosulfate (PICO) with magnesium citrate (Picosolution, Pambio Co, Seoul Korea).

**4. STUDY METHODS**

**4.1 Study design**

This was a multicenter, randomized controlled, non-inferiority study. Patients were randomized into a 1 L PEG with ascorbic acid group and a PICO with magnesium citrate group according to the bowel cleansing agent used.

**4.2 Study period**

24 months from the date of Institutional Review Board (IRB) approval.

**4.3 Study subjects**

**4.3.1 Inclusion criteria**

- Outpatients aged between 19 and 75 years old.

- Those who are scheduled to undergo colonoscopy for diagnosis or follow-up surveillance.

- Those who voluntarily decided to participate in this clinical trial and gave their written consent.

**4.3.2 Exclusion criteria**

- Patients with a history of gastrointestinal tract surgery or acute abdominal conditions requiring surgery.

-Patients with inflammatory bowel disease, intestinal obstruction, or pseudo-obstruction.

- Patients with impaired renal function, pregnancy, recent malignancy.

- Patients with severe constipation, taking laxatives or gastrointestinal motility drugs within the past three months.

- Patients who have diagnosed with colon polyps and have been transferred for removal.

-Patients who did not consent to the study.

**4.4 Detailed study method**

The 1 L PEG with ascorbic acid group and PICO with magnesium citrate group are assigned in a 1:1 ratio using computer-generated randomization with patients who agreed and were suitable for this study. Both groups should be on a low-residue diet from 3 days before and eat white rice porridge the day before the trial. (Supplementary appendix 1) Each group administered as a split-dose regimen according to domestic approval. (Supplementary appendix 2,3) Bowel preparation for study is educated by a professional nurse with a same printout in all hospitals. Have all patients fill out a questionnaire on the day of colonoscopy. Colonoscopy performed as a single-blinded in which the endoscopist had no information about any bowel cleansing agent.


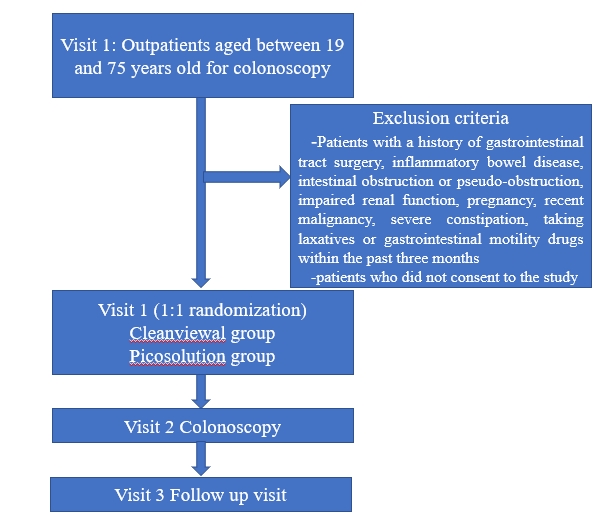


**4.4.1 Main analysis**

- The success rate of bowel preparation between the two groups through the Harefield cleansing scale.

- The rate of high-quality bowel cleansing through the Harefield cleansing scale.

- Patient satisfaction and compliance through the patient outcome questionnaire.

- Safety through the patient outcome questionnaire about adverse effect.

**4.4.2 Other observation**

- Basic personal information (Gender, age, weight, comorbidities, previous history of taking bowel cleansing agent).

- Colonoscopy information (Indication, finding, intubation time, procedure time).

**4.5 Outcome variables**

**4.5.1 Primary outcome variables**

1. the success rate of bowel preparation through the Harefield cleansing scale (HCS)

-Success of bowel preparation: A or B

-Fail of bowel preparation: C or D

-Harefield Cleaning Scale

**4.5.2 Secondary outcome variables**

1. Harefield cleansing scale for each segment of colon.

2. Adenoma detection rate.

3. Patient satisfaction and acceptability (Taste, feel, ease of taking).

4. Adverse effect (Nausea, vomiting, abdominal pain, abdominal distension, insomnia, and paresthesia).

**4.6 Sample size**

The appropriate sample size was calculated based on a previous phase 3 trial.^11^ The ratio between the 1 L PEG with ascorbic acid group and the PICO with magnesium citrate group was assumed to be 1:1, and the expected treatment rate (effectiveness) of the test drug and the comparator drug was P1 (1 L PEG) =0.83, P2 (PICO) =0.95, significance level α=0.05, power 80%. The minimum number of patients required for the clinical trial was 117 for each group. Considering a dropout rate of approximately 10% in each group, there were 127 subjects in each group, for a total of 254 subjects. The number of patients required for the study was calculated as follows by using the G-power 3.1.9.7 program.

**4.7 Analysis Plan (Including statistical methods)**

Continuous variables are reported as mean (standard deviation, SD), and discrete data are expressed as numbers and percentages. Categorical data were analyzed using frequency (%) analyses, the χ2 test, and Fisher’s exact test, and quantitative data were analyzed using the independent sample t-test. The HCS of the 1 L PEG with ascorbic acid group was compared with that of the PICO with magnesium citrate group using a non-inferiority test. Non-inferiority was confirmed only if the one-sided 97.5% lower confidence limit for the difference between treatments was ≥-10. Statistical significance was defined as p<0.05. All statistical analyses were performed using SPSS Statistics version 26.0 (IBM Corp., Armonk, NY, USA).

**4.8 Study timeline**

| IRB approval ~ 24 months | | | | | | | | | | | | |
| --- | --- | --- | --- | --- | --- | --- | --- | --- | --- | --- | --- | --- |
| Months | 2 | 4 | 6 | 8 | 10 | 12 | 14 | 16 | 18 | 20 | 22 | 24 |
| 1. IRB acquisition 2. Data Collection 3. Result analysis and thesis writing | O | O | O | O | O | O | O | O | O | O | O | O |

**5. CRITERIA FOR STUDY DICONTINUATION AND WITHDRAWAL**

- When the patient withdraws consent.

- In case clinical information is not entered in the case record.

- When the clinical investigator decides that the study should be stopped.

**6. PREDICTED ADVERSE EFFECT OF DRUG AND PRECAUTION**

**6.1 Predicted adverse effect**

In this clinical trial, adverse effect refers to all unpredictable medical problems that may occur during the clinical trial. If adverse effects that require hospitalization, such as convulsions, severe dehydration, and loss of consciousness, occur after taking bowel preparation, the investigators must immediately report to the IRB and the Clinical Trial headquarters. In a recent multicenter study, they analysed the difference between the low-dose laxative group and the PEG laxative, including elderly patients up to 80 years of age, and no statistical difference was observed in safety.^7,8^ Therefore, the predicted adverse effects are also expected to be within the range of adverse effects of the existing bowel preparations that are being implemented in clinical practice.

**-** Vomiting, abdominal distension, abdominal pain, dizziness may occur while taking or after taking all the bowel preparation. These adverse effects are predictable and, in most cases, resolve spontaneously. Cases of hospitalization due to convulsions or loss of consciousness have been reported very rarely, but recovery was possible with conservative treatment in most cases. Nephrotoxicity is reported to occur in about 5% of cases, and it is expected to be similar in both groups. ^9,10^ Most nephrotoxicity improves with conservative treatment such as hydration.

- When a colonoscopy performed after taking a bowel preparation, the colonoscopy may be re-examined due to poor bowel preparation. analysing the cases of poor bowel preparation, most of the factors are related to inability to take the preparation agent or the food eaten before the colonoscopy. Therefore, for proper bowel preparation, it is important for the patient to follow the precautions for taking the bowel preparation and to take the preparation as directed by the medical staff.

**-** Other predictable side effects include bleeding and perforation as complications that can occur when undergoing colonoscopy or polypectomy. However, these complications are due to colonoscopic procedure and are not complications due to the use of bowel preparations. It is difficult to call these complications related to this study.

**6.2 How to report adverse effect.**

If it is determined that it is an adverse effect, contact the clinical trial adverse effect monitor.

- Kyu-won Kim (Clinical research coordinator).

- Address: Chosun university hospital, 365, Pilmun-daero, Dong-gu, Gwagnju, 61453, Republic of Korea.

- Telephone: +82-62-220-3012.

**7. SIGNIFICANCE AND IMPACT**

This study will provide information on efficacy and safety of 1 L PEG with ascorbic acid and safety and will contribute to broadening the options for bowel preparation.

8. REFERENCES

1. Jung KW, Won YJ, Kong HJ, et al. Cancer statistics in Korea: incidence, mortality, survival, prevalence in 2012. Cancer Res Treat 2015;47:127-41.

2. Brenner H, Chang-Claude J, Seiler CM, et al. Protection from colorectal cancer after colonoscopy: a population-based, case-control study. Ann Intern Med 2011;154:22-30.

3. Nishihara R, Wu K, Lochhead P, et al. Long-term colorectal-cancer incidence and mortality after lower endoscopy. N Engl J Med 2013;369:1095-105

4. Arain MA, Sawhney M, Sheikh S, et al. CIMP status of interval colon cancers: another piece to the puzzle. Am J Gastroenterol 2010;105:1189-95.

5. Rex DK, Imperiale TF, Latinovich DR, et al. Impact of bowel preparation on efficiency and cost of colonoscopy. Am J Gastroenterol 2002;97:1696-700.

6. Harewood GC, Sharma VK, de Garmo P. Impact of colonoscopy preparation quality on detection of suspected colonic neoplasia. Gastrointest Endosc 2003;58:76-9.

7. Lukens FJ, Loeb DS, Machicao VI, et al. Colonoscopy in octogenarians: a prospective outpatient study. *Am J Gastroenterol*. 2002;97:1722-1725.

8. Bat L, Pines A, Shemesh E, et al. Colonoscopy in patients aged 80 years or older and its contribution to the evaluation of rectal bleeding. *Postgrad Med J*. 1992;68:355-358.

9. Hurst FP, Bohen EM, Osgard EM, et al. Association of oral sodium phosphate purgative use with acute kidney injury. *J Am Soc Nephrol*. 2007;18:3192-3198.

10. Abaskharoun R, Depew W, Vanner S. Changes in renal function following administration of oral sodium phosphate or polyethylene glycol for colon cleansing before colonoscopy. *Can J Gastroenterol*. 2007;21:227-231.

11. Schreiber S, Baumgart DC, Drenth JPH, Filip RS, Clayton LB, Hylands K, et al. Colon cleansing efficacy and safety with 1 L NER1006 versus sodium picosulfate with magnesium citrate: a randomized phase 3 trial. Endoscopy. 2019;51(1):73-84
