# Supplementary material for: Comparison of optimal bowel cleansing effects of 1L polyethylene glycol with ascorbic acid versus sodium picosulfate with magnesium citrate: A randomized controlled study
Source: PLoS One. 2022 Dec 30;17(12):e0279631. doi: 10.1371/journal.pone.0279631 (PMC9803231; doi:10.1371/journal.pone.0279631)
Supplement: S2 File — (ZIP) [file pone.0279631.s003.zip › Study protcol, statement, consent form, consort check list/Full-lenth protcol.(Korean).docx]

**대장내시경 전처치 하제로 1L ascorbic acid 함유 PEG 제제와 magnesium citrate 함유 피코솔루션 제제의 효과와 안정성에 대한 임상 연구**

**Randomized clinical trial on efficacy and safety of 1L polyethylene glycol with ascorbic acid versus sodium picosulfate with magnesium citrate as preparation for colonoscopy**

**책임연구자: 소화기내과 이준**

**목차**

**1.연구제목 ---------------------------------------------------------------------------------------- 1**

**2.연구과제 단계 --------------------------------------------------------------------------------- 1**

**3.연구실시기관명 및 주소----------------------------------------------------------------------- 1**

**4.연구책임자 및 담당자 ------------------------------------------------------------------------- 1**

**5.연구배경------------------------------------------------------------------------------------------2**

**6.연구목적------------------------------------------------------------------------------------------2**

**7.예상연구기간 ------------------------------------------------------------------------------------2**

**8.연구대상--------------------------------------------------------------------------------------- 3**

**9.연구방법-----------------------------------------------------------------------------------------4**

**10.예측 부작용 및 주의사항----------------------------------------------------------------------5**

**11.중지 및 탈락 기준---------------------------------------------------------------------------- 5**

**12.부작용을 포함한 안전성의 평가기준, 평가방법 및 보고방법 -------------------------------6**

**13.취약한 환경에 있는 대상자 동의 -------------------------------------------------------------8**

**14.피해자 보상에 대한 규약 ------------------------------------------------------------------- 8**

**15.통계 방법 ------------------------------------------------------------------------------------ 9**

**16.자료 관리 계획 및 개인정보보안 ----------------------------------------------------------- 9**

**17.연구비 지출계획 ----------------------------------------------------------------------------- 9**

**18.참고문헌 --------------------------------------------------------------------------------------10**

**1. 연구제목**

국문: 대장내시경 전처치 하제로 1L ascorbic acid 함유 PEG 제제와 magnesium citrate 함유 피코솔루션 제제의 효과와 안정성에 대한 임상 연구

영문: Randomized clinical trial on efficacy and safety of 1L polyethylene glycol with ascorbic acid versus sodium picosulfate with magnesium citrate as preparation for colonoscopy

**2. 연구과제 단계**

연구자 주도 전향적 임상연구

**3. 연구실시 기관명 및 주소, 연구비 지원기관**

**1) 연구기관**

조선대학교병원 : 61453. 광주광역시 동구 필문대로 365.

전남대학교병원 :61469 광주광역시 동구 학동 제봉로 42

화순전남대학교병원 : 58128 전남 화순군 화순읍 서양로 322

원광대학교병원 : 54538 전북 익산시 무왕로 895

**2) 연구비 지원기관 명 및 주소(모니터 직명 및 성명 포함)**
 ㈜ 태준 제약: 서울특별시 용산구 대사관로 31길 8.

**4. 연구책임자 및 담당자**

**1) 연구 책임자**

본원 연구책임자: 조선대학교 병원 소화기내과 교수 이준

본원 연구담당자: 조산대학교 병원 소화기내과 전임의 김성중

**2) 공동 연구자**

전남대학교 병원 소화기내과 교수 김현수
화순전남대학교병원 소화기내과 교수 주영은
전북대학교 병원 소화기내과 교수 김상욱

원광대학교 병원 소화기내과 교수 서검석

**5. 연구배경**

우리나라 대장암 발생율은 인구 10만 명 당 남자에서 69.3명, 여자에서 45.9명으로, 갑상선과 위암 다음으로 세 번째로 호발하는 암이다.^1^ 대장암의 80% 이상이 선종-암화 과정으로 진행되므로 대장내시경을 시행하여 선종을 제거하는 것은 대장암의 발생율과 사망률을 현저히 낮출 수 있다.^2,3^. 그러나 대장내시경을 적절한 간격으로 시행했음에도 불구하고 6-8% 정도 중간암 발생이 보고되고 있다.^3,4^ 불충분한 장정결은 선종을 미발견율을 높이고, 시술시간이 길어지며, 비용과 시간의 손실을 초래하고, 궁긍적으로 중간암의 주요한 원인이 될 수 있다.^5,6^ 이상적인 장정결제는 높은 효능이 있어야 하고, 안정성이 확보되어야 하며, 복약순응도가 높아야 한다. 현재 이용할 수 있는 장정결제는 기존의 polyethylene glycol (PEG) 4L, 다양한 저용량하제(PEG plus ascorbate 2L, oral sulfated solution(OSS), picosulfate plus magnesium citrate)등이 있다. 저용량 하제는 기존의 4L PEG에 비해 하제의 용량을 절반으로 줄였지만, 다량의 물을 복용해야 하는 단점이 있다. 최근 기존의 PEG에 ascorbic acid의 함량을 높여 하제의 용량을 기존의 저용량하제보다 절반으로 감소시킨 초저용량하제인 1L PEG plus ascorbic acid 제제가 새롭게 출시되었다. 초저용량하제인 1L PEG plus ascorbic acid는 3상 연구를 통해 기존의 2L PEG plus ascorbate에 비교하여 장정결의 효과와 환자의 재복용 선호도, 그리고 부작용 측면에 유사한 결과를 확인하였다. 그러나 현재까지 1L PEG plus ascorbic acid와 다른 저용량 하제와의 비교 연구가 부족한 실정이다.

**6. 연구목적**

본 연구는 초저용량하제(1L)와 기존의 저용량하제를 비교 분석하는 연구이다.

대장내시경 검사 시의 전처지용 장세척을 예정하는 성인을 대상으로 **1L PEG plus ascorbic acid(Cleanviewal powder, Taejoon Pharm. Co, Seoul Korea. 크린뷰올산, 태준제약)**와 **picosulfate plus magnesium citrate(Picosolution, Pambio Co, Seoul Korea, 피코솔루션, 팜비오제약)**을 투여한 후 장정결의 효과, 안정성, 순응도, 선호도 및 만족도를 평가하고자 한다.

**7. 예상연구기간**

○IRB 승인일 ~ 승인일로부터 24개월

| ( 연구기간 : IRB 승인일  - 24개월 ) | | | | | | | | | | | | |
| --- | --- | --- | --- | --- | --- | --- | --- | --- | --- | --- | --- | --- |
| 月 | 1 | 2 | 3 | 4 | 5 | 6 | 7 | 8 | 9 | 10 | 11 | 12 |
| 1. IRB 심의 2. 자료 분석 3. 자료 취합 및 분석 4. 결과 및 논문 작성 | O | O | O | O | O  O | O | O | O | O  O | O | O | O |

**8. 연구대상**

**8.1. 연구대상**

등록된 각 기관의 병원에서 대장내시경을 시행 예정인 환자를 대상으로 본 임상시험의 선정기준에 합당하고 제외기준에 하나도 해당되지 않는 환자를 대상으로 한다.

**8.2 연구대상자 선정기준**

1. 연구 동의일 기준으로 만 19이상 75세 미만 남녀
2. 진단 또는 검진, 추적 검사 목적으로 대장내시경 검사가 예정된 자
3. 본 임상시험에 자의로 참여를 결정하고 서면 동의한 자
   1. **연구대상자 제외기준**
4. 장절제술이나 위절제술과 같은 위장관 절제 수술을 받은 환자 또는 외과수술이 필요한 급성복부 상태(예; 급성 충수염)
5. 장폐색, 위장관 폐색, 위장관 궤양, 위마비, 염증성 장질환이 있거나 의심되는 자
6. 중증의 신장애, 심부전, 복수 등 심각한 내과적 기저 질환이 있는 자.
7. 복용할 장정결제나 그 성분에 과민반응이 있는 자.
8. 활성기 암환자
9. 심한 변비가 있거나, 최근 3개월이내에 변비약이나, 위장관 운동 약물을 먹고 있는 자.
10. 대장 용종이나 종양을 진단받고 제거 위해 전원된 자
11. 피험자 동의서에 서명을 거부한 환자

**8.4 목표한 연구대상자의 수 및 그 근거**

기존 연구들을 바탕으로 각각의 하제가 적절한 장결도를 보이는 비율이 90%라고 가정하고 양 군의 차이가 10%이상 차이가 날 경우, 통계적으로 비열등하다고 정의하였고, 통계력 지수 0.8로, 탈락율을 10%를 고려하면 각 군당 124명이고, 최소한 248명의 수검자가 필요하다. 각 병원당 50명(시험군, 대조군 각 25명) 250명을 목표로 한다.

**9. 연구방법**

연구대상자 선정기준에 합당한 환자를 대상으로 1:1 무작위 배정을 통해 대장내시경 시행전 장정결제를 기준으로 시험군(크린뷰올산군)과 대조군(피코솔루션투여군)으로 할당한다.

두군 모두 3일전부터 저잔류식사(low-residue diet)를 하고 검사 전날은 흰쌀죽으로 식사한다.(첨부 1. 대장내시경을 위한 주의사항) 각 군은 분할요법(split-dose regimen)으로 국내 허가사항 대로 복용한다.(첨부 2.3 복용안내문 1(크린뷰올산), 2(피코솔루션)) 검사 전 장정결제 복용 교육은 5개병원에 모두 공통적인 서식(인쇄문)을 통해 일반적인 환자와 유사하게, 전문간호사가 말과 지면 2가지방법 모두 설명한다(verbal and written instruction) 대장내시경 시행 당일 모든 수검자들로 하여금 설문지를 작성하게 한다. 대장내시경 시행의사는 정결방법에 대해서 단일 눈가림으로 진행된다. 시술 전후 합병증 발생에 대해 집중적으로 확인한다.

**9.1 기본적인 인구 역동학적 조사**

나이, 성별, 체질량지수, 기저 질환, 대장내시경 시행 적응증, 삽입시간, 시술시간 등을 확인한다.

**9.2 주 평가 변수(primary endpoint)**

장정결도 평가지수(Harefield Cleansing Scale)척도상 성공비율
(성공: A or B, 실패: C or D)
 - Harefield 장정결도 척도상 A 또는 B에 해당하는 대상자 비율.

참고) Harefield Cleaning Scale(HCS)

- 1. **부 평가 변수(Secondary endpoint)**

1. 각 구획 별 Harefiled 장정결도 점수 비율
2. 선종발견율
3. 환자 순응도(완전복용여부, 맛, 양, 재복용여부 등)
   a. 장정결 복용에 대한 느낌
   b. 장정결제의 맛
   c. 장정결제에 대한 만족도.
   c. 재 복용 의사
4. 안전성 평가(구토, 구역, 복통, 등 부작용 확인: 검사당일과 1주일 뒤 외래)
   a. 소화기계 부작용
    :구역, 구토, 복통, 탈수(목마름 등)
   b. 다른 부작용
    : 수면 장애, 두통, 항문소양감, 경련

**9.4 연구 계획 모식도**

**

10. 예측 부작용 및 주의사항**

대장내시경 시행 전 장정결제 복용으로 인해 드물게 탈수, 전해질 이상, 경련, 무기력증이 나타날 수 있다. 또한 오심과 구토를 인한 장정결제 복용 실패할 수 있다.
전반적인 부작용 평가 기준, 방법, 보고방법은 12번 항목에 자세히 기술함.

**11. 중지 및 탈락 기준**

1. 본 연구의 중지 및 탈락 기준은 환자가 동의를 철회한 경우이다.
2. 증례기록지의 형식에 부합한 정보의 기입이 이루어지지 않는 경우
3. 기타 임상시험담당자가 비교연구를 중지하여야 한다고 판단한 경우

**12. 부작용을 포함한 안전성의 평가기준, 평가방법 및 보고방법**

1) 부작용의 평가 기준 및 방법

본 임상시험에서 부작용이라 함은 임상시험 도중 발생할지 모른 예측하지 못한 모든 의학적 문제를 의미한다. 장정결제 복용 후 경련, 중증 탈수 및 의식 감소 등의 입원을 필요로 하는 부작용이 발생할 경우에는 담당 연구진은 즉시 IRB와 임상시험 본부로 수기 보고해야 하며 부작용발생과 관련하여 원인 및 대처에 대하여 책임연구자와 공동연구자들의 검토를 거쳐 연구대상자 제외, 연구기관 제외 혹은 연구 종결을 결장한다. 최근 외국에서 시행된 다기관 연구에서, 80세까지의 고령환자들을 포함하여 저용량 하제 사용군과 PEG하제와의 차이를 분석하였는데 통계학적 차이는 관찰되지 않았다.^7,8^ 따라서, 예측되는 부작용/합병증도 임상에서 시행되고 있는 두 군의 장정결제의 기존 부작용/합병증 부작용 범위 이내일 것으로 예상한다. 본 연구에서 진행하는 시술 전 장정결제 사용은 본 연구 목적이 아니더라도 대장내시경 검사를 위해서는 필수적인 전처치이다. PEG 및 OSS 모두 임상현장에서 이미 사용되고 있는 장정결제이기 때문에 연구로 인해 추가적인 위험이 더 가해질 가능성은 없다.

1. 장정결제를 복용하는 중 혹은 모두 복용한 후에 구토, 복부팽만, 복통, 어지러움증 등이 발생할 수 있으며 드물게는 구갈, 감각 이상, 손발저림, 경련, 의식저하 등의 부작용이 있을 수 있다. 이러한 부작용은 예측이 가능하며 대부분 자연 호전된다. 경련이나 의식저하 등으로 입원 치료를 하였던 경우도 아주 드물게 보고되고 있지만 대부분 보존적인 치료로 회복이 가능하였으며 이러한 장정결과 연관된 증상이 발생할 시에는 원인 교정을 위해 혈액검사를 시행하고 입원 등 치료를 위한 필요한 조치를 취하게 된다. 신독성은 일반적으로 5% 전후에서 발생하는 것으로 보고되고 있고^9-10^ 두 군 모두에서 유사할 것으로 예측한다. 대부분 수액 공급 등 보존 치료로 호전된다.

② 장정결제를 복용한 후 대장내시경을 시행하였을 때 장정결이 불량하여 대장내시경 재 검사를 받을 수 있다. 불량한 정결상태를 보였던 경우를 분석해 보면 약제를 끝까지 다 복용하지 못하거나 대장내시경 시행 이전 섭취한 음식과 연관이 많아 대부분 수검자와 관련된 요인이 많다. 따라서 적합한 장정결 상태를 보이기 위해서는 장정결제 복용과 관련된 주의 사항을 잘 지키고 의료진의 지시대로 성실하게 정결제를 복용하는 것이 중요하다.

③ 그 이외에 예측 가능한 부작용들은 대장내시경을 시행 받거나 대장폴립절제술을 시행 받을 시에 발생할 수 있는 시술 합병증으로서 출혈, 천공 등이 있다. 하지만, 이런 합병증들은 대장내시경 시술 자체에 의한 합병증이지 장정결제 복용에 의한 합병증은 아니기 때문에 본 연구와 관련된 합병증들이라고 하기 어렵다.

2) 부작용 보고 방법

임상시험 부작용 모니터

이 름: 김규원 (연구간호사)

주 소: 광주광역시 동구 필문대로 365 조선대병원 소화기내과

전 화: 062-220-3012

**13. 취약한 환경에 있는 대상자 동의**

시험기관의 IRB의 의견에 따라 취약한 환경에 있는 시험대상자(예: 원내 직원, 치매 등 동의 능력이 손상된 사람 등)를 모집할 수 있으며, 시험책임자 및 시험담당자는 취약한 환경에 있는 시험대상자 등록 시 아래와 같은 방안과 시험자의 의무를 준수하여야 한다. 단, 시험기관의 IRB에서 취약한 환경에 있는 시험대상자의 모집을 승인하지 아니한 경우에는 등록 할 수 없다.

1) 시험책임자 또는 시험책임자의 위임을 받은 사람은 심사위원회의 승인을 받은 서면 정보와 그 밖에 임상시험의 모든 측면에 대한 정보를 대상자에게 충분히 알려야 한다. 이 경우 대상자가 동의할 수 없는 경우(예: 치매 등 동의 능력이 손상된 사람)에는 대상자의 대리인에게 이를 알려야 한다.

- 대상자 또는 대상자의 대리인이 동의서 서식, 시험대상자설명서, 그 밖의 문서화된 정보를 읽을 수 없는 경우에는 참관인이 동의를 받는 모든 과정에 참석하여야 한다. 이 경우 시험책임자 또는 시험책임자의 위임을 받은 자는 동의서 서식, 시험대상자설명서, 그 밖의 문서화된 정보를 대상자 또는 대상자의 대리인에게 읽어 주고 설명하여야 하며, 대상자 또는 대상자의 대리인은 대상자의 임상시험 참여를 말로 동의하고 가능하면 동의서에 자필로 서명하고 해당 날짜를 적고, 참관인이 동의서에 자필로 서명하고 해당 날짜를 적어야 한다.

2) 취약한 환경에 있는 대상자 등록 시 시험자의 의무

- 임상시험 참여 여부에 대해 자유롭게 자발적으로 선택할 수 있도록 하여야 한다.

- 어떠한 형태로든 임상시험 참여를 강요해서는 안된다.

- 언제든지 임상시험 참여를 철회 할 수 있도록 하여야 한다.

- 임상시험 참여 여부와 관련하여 어떠한 불이익이 없도록 하여야 한다.

**14. 피해자 보상에 대한 규약**

연구책임자는 본 임상연구 실시에 대하여 다음 사실을 확인해야 한다.

- 본 연구 실시 중에 본 연구로 인해 이상반응 등 예기치 않은 사고 등이 발생하여 이에 대한 치료 또는 입원이 요구되거나 피험자 또는 보고자와 분쟁이 발생하는 경우에는 연구자가 그 비용을 부담한다.

- 이러한 이상반응 처리는 다음 사항에 적합하여야 함.

첫째, 책임자 및 담당자가 본 임상연구계획서 내용을 충실히 이행하여야 하며

둘째, 본 시험 실시에 대한 책임자 및 담당자의 태만이나 의도적 또는 중대한 과실이 인정되지 말아야 하며

셋째, 발생한 이상반응에 대하여는 연구자에게 즉각적인 연락을 취하여 이에 대한 준비를 하도록 하여야 한다.

- 다만, 부작용이 발생하였을 경우에도 임상시험과 무관하게 대장내시경 검사 자체에 의한 부작용으로 판단되었을 때에는 보상하지 않는다.

**15. 통계 방법**

모든 분석은 statistical software (SPSS)를 통하여 시행하며, 각 군의 장정결도 비교에서의 수치형 변수는 t-test를 시행하여 평균과 표준편차로 표시하고 범주형 변수는 chi-square test를 이용하여 분석한다. 모든 검정에는 p값이 0.05미만인 경우를 의미 있는 것으로 판정한다.

**16. 자료 관리 계획 및 개인정보보안**

자료 수집 시 환자에 대한 정보는 최소화하여 직접적인 정보 유출은 없을 것으로 판단되며 환자 이름과 주민번호는 명시되지 않을 것이며 환자 등록번호는 암호화 할 것이며, 환자 정보가 유출되지 않도록 환자 정보 보호를 할 것이다. 환자 정보에 대한 접근은 연구책임자 및 공동연구자로 제한할 것이며 환자 정보 file은 암호화하여 잠금 장치에 보관하고 접근이 제한된 컴퓨터에 저장, 접근 암호를 제한하고 책임연구자만 접근 가능하도록 할 것이다. 모든 정보는 연구가 종료 후 3년간 보관 후 폐기될 것이다.

.

**17. 연구비지출계획**

첨부파일(서식 2호) 참조.

참고문헌

1. Jung KW, Won YJ, Kong HJ, et al. Cancer statistics in Korea: incidence, mortality, survival, and prevalence in 2012. Cancer Res Treat 2015;47:127-41.

2. Brenner H, Chang-Claude J, Seiler CM, et al. Protection from colorectal cancer after colonoscopy: a population-based, case-control study. Ann Intern Med 2011;154:22-30.

3. Nishihara R, Wu K, Lochhead P, et al. Long-term colorectal-cancer incidence and mortality after lower endoscopy. N Engl J Med 2013;369:1095-105

4. Arain MA, Sawhney M, Sheikh S, et al. CIMP status of interval colon cancers: another piece to the puzzle. Am J Gastroenterol 2010;105:1189-95.

5. Rex DK, Imperiale TF, Latinovich DR, et al. Impact of bowel preparation on efficiency and cost of colonoscopy. Am J Gastroenterol 2002;97:1696-700.

6. Harewood GC, Sharma VK, de Garmo P. Impact of colonoscopy preparation quality on detection of suspected colonic neoplasia. Gastrointest Endosc 2003;58:76-9.

7. Lukens FJ, Loeb DS, Machicao VI, et al. Colonoscopy in octogenarians: a prospective outpatient study. *Am J Gastroenterol*. 2002;97:1722-1725.

8. Bat L, Pines A, Shemesh E, et al. Colonoscopy in patients aged 80 years or older and its contribution to the evaluation of rectal bleeding. *Postgrad Med J*. 1992;68:355-358.

9. Hurst FP, Bohen EM, Osgard EM, et al. Association of oral sodium phosphate purgative use with acute kidney injury. *J Am Soc Nephrol*. 2007;18:3192-3198.

10. Abaskharoun R, Depew W, Vanner S. Changes in renal function following administration of oral sodium phosphate or polyethylene glycol for colon cleansing before colonoscopy. *Can J Gastroenterol*. 2007;21:227-231.
