# Supplementary material for: Comparison of optimal bowel cleansing effects of 1L polyethylene glycol with ascorbic acid versus sodium picosulfate with magnesium citrate: A randomized controlled study
Source: PLoS One. 2022 Dec 30;17(12):e0279631. doi: 10.1371/journal.pone.0279631 (PMC9803231; doi:10.1371/journal.pone.0279631)
Supplement: S2 File — (ZIP) [file pone.0279631.s003.zip › Study protcol, statement, consent form, consort check list/Statement for consent of clinical trial subject(Korean).docx]

**[서식 10]**

**연구대상자 설명문**

**연구과제명 : 대장내시경 전처치 하제로 1L 아스코빅산(ascorbic acid) 함유 폴리에틸렌글리콜(PEG) 제제와 마그네슘 시트레이트 함유 피코솔루션 제제의 효과와 안전성에 대한 임상연구**

본 연구는 대장내시경 전처치 하제로 **대장내시경 전처치 하제로 1L 아스코빅산(ascorbic acid) 함유 폴리에틸렌글리콜(PEG) 제제와 마그네슘 시트레이트 함유 피코솔루션 제제의 장정결 효과, 복용 순응도, 안정성에 대한** 연구입니다. 본 임상시험은 연구목적을 가진 연구이므로 검증되지 않은 실험적인 측면이 있습니다. 귀하는 본 연구에 참여할 것인지 여부를 결정하기 전에, 설문서와 동의서를 신중하게 읽어보셔야 합니다. 이 연구가 왜 수행되며, 무엇을 수행하는지 귀하가 이해하는 것이 중요합니다. 이 연구를 수행하는 ___이 준___연구책임자(김규원 연구 간호사 또는 _김성중 연구담당자)가 귀하에게 이 연구에 대해 설명해 줄 것입니다. 이 연구는 자발적으로 참여 의사를 밝히신 분에 한하여 수행 될 것입니다. 다음 내용을 신중히 읽어보신 후 참여 의사를 밝혀 주시길 바라며, 필요하다면 가족이나 친구들과 의논하시기 바라며, 의논하실 분이 없으신 경우 조선대학교병원 기관생명윤리위원회에 공정한 입회자를 요청할 수 있습니다. 담당 연구원은 이 연구에 관한 귀하의 어떠한 질문에 대해서도 자세하게 설명해 줄 것입니다. 귀하의 서명은 귀하가 본 연구에 대해 그리고 위험성에 대해 설명을 들었으며, 귀하께서 자신(또는 법정대리인)이 본 연구에 참가를 원한다는 것을 의미합니다.

**1. 연구의 배경과 목적**

우리나라 대장암 발생율은 인구 10만 명 당 남자에서 69.3명, 여자에서 45.9명으로, 갑상선과 위암 다음으로 세 번째로 호발하는 암입니다. 대장암의 80% 이상이 선종-암화 과정으로 진행되므로 대장내시경을 시행하여 선종을 제거하는 것은 대장암의 발생율과 사망률을 현저히 낮출 수 있습니다. 그러나 대장내시경을 적절한 간격으로 시행했음에도 불구하고 6-8% 정도 중간암 발생이 보고되고 있습니다. 불충분한 장정결은 선종을 미발견율을 높이고, 시술시간이 길어지며, 비용과 시간의 손실을 초래하고, 궁긍적으로 중간암의 주요한 원인이 될 수 있습니다. 이상적인 장정결제는 높은 효능이 있어야 하고, 안정성이 확보되어야 하며, 복약순응도가 높아야 합니다. 기존의 저용량 하제는 기존의 4L 폴리에틸렌글리콜에 비해 하제의 용량을 절반으로 줄였지만, 다량의 물을 복용해야 하는 단점이 있습니다. 최근 기존의 폴리에틸렌글리콜에 아스코빅산의 함량을 높여 하제의 용량을 기존의 저용량하제보다 절반으로 감소시킨 초저용량하제인 1L PEG plus ascorbic acid 제제가 새롭게 출시되었습니다. 초저용량하제인 1L 아스코빅산(ascorbic acid) 함유 폴리에틸렌글리콜(PEG) 제제는 3상 연구를 통해 기존의 2L 저용량하제(아스코빅산 함유 폴리에틸렌클리콜)에 비교하여 장정결의 효과와 환자의 재복용 선호도, 그리고 부작용 측면에 유사한 결과를 확인하였습니다. 그러나 현재까지 1L 아스코빅산(ascorbic acid) 함유 폴리에틸렌글리콜(PEG) 제제와 다른 저용량 하제와의 비교 연구가 부족한 실정입니다. 본 연구는 초저용량하제(1L)와 기존의 저용량하제를 비교 분석하는 연구입니다. 대장내시경 검사 시의 전처지용 장세척을 예정하는 성인을 대상으로 1L 1L 아스코빅산(ascorbic acid) 함유 폴리에틸렌글리콜(PEG) 제제(Cleanviewal powder, Taejoon Pharm. Co, Seoul Korea. 크린뷰올산, 태준제약)와 마그네슘 시트레이트 함유 피코솔루션 제제(Picosolution, Pambio Co, Seoul Korea, 피코솔루션, 팜비오제약)을 투여한 후 장정결의 효과, 안정성, 순응도, 선호도 및 만족도를 평가하고자합니다.

**2. 연구 참여 대상자수**

이 연구는 본 기관을 포함하여 4개 대학병원에서 250명을 목표로 연구대상자가 참여하게 됩니다.

**3. 연구 방법 및 예측 결과(효과)**

이 연구는 전향적으로 이루어지지만 연구를 위하여 실제 검사 방법과 다른 형태를 취하지는 않으며, 본 연구가 귀하의 현재 또는 향후 검사 및 치료에 전혀 영향을 미치지 않습니다. 귀하는 **1L 아스코빅산(ascorbic acid) 함유 폴리에틸렌글리콜(PEG) 제제 또는 마그네슘 시트레이트 함유 피코솔루션 제제**를 이용하여 장정결을 하게 됩니다. 대장내시경 검사 및 치료는 일반적인 검사와 동일하지만, 귀하가 저희 병원에서 진단과 치료를 위해 시행하였던 내시경 검사를 포함한 의무 기록을 저희가 열람하고, 그 중 일부가 연구 목적으로 논문에 인용될 수 있습니다. 하지만 연구 목적으로 논문에 인용되는 정보가 있다고 하더라도 환자의 개인정보는 철저히 보호될 것입니다.

**4. 연구 참여 기간**

본 시험의 전체 연구기간은 12개월로 예상됩니다.(승인일부터 1년간)

**5. 연구 참여 도중 중도탈락**

귀하는 연구에 참여하신 후에도 언제든지 도중에 그만 둘 수 있습니다. 만일 귀하가 연구에 참여하는 것을 그만두고 싶다면 담당 연구원이나 연구책임자에게 즉시 말씀해 주십시오.

**6. 연구 참여로 인해 발생 가능한 부작용(위험요소)**

1) 부작용의 평가 기준 및 방법

본 임상시험에서 부작용이라 함은 임상시험 도중 발생할지 모른 예측하지 못한 모든 의학적 문제를 의미합니다. 장정결제 복용 후 경련, 중증 탈수 및 의식 감소 등의 입원을 필요로 하는 부작용이 발생할 경우에는 담당 연구진은 즉시 IRB와 임상시험 본부로 수기 보고해야 하며 부작용발생과 관련하여 원인 및 대처에 대하여 책임연구자와 공동연구자들의 검토를 거쳐 연구대상자 제외, 연구기관 제외 혹은 연구 종결을 결장합니다. 최근 외국에서 시행된 다기관 연구에서, 80세까지의 고령환자들을 포함하여 저용량 하제 사용군과 PEG하제와의 차이를 분석하였는데 통계학적 차이는 관찰되지 않았습니다. 따라서, 예측되는 부작용/합병증도 임상에서 시행되고 있는 두 군의 장정결제의 기존 부작용/합병증 부작용 범위 이내일 것으로 예상됩니다. 본 연구에서 진행하는 시술 전 장정결제 사용은 현재 우리나라에서 대장내시경 검사를 위해서 허가가 되어있는 필수적인 전처치입니다. **1L 아스코빅산(ascorbic acid) 함유 폴리에틸렌글리콜(PEG) 제제 또는 마그네슘 시트레이트 함유 피코솔루션 제제** 모두 임상현장에서 이미 사용되고 있는 장정결제이기 때문에 연구로 인해 추가적인 위험이 더 가해질 가능성은 없습니다.

① 장정결제를 복용하는 중 혹은 모두 복용한 후에 구토, 복부팽만, 복통, 어지러움증 등이 발생할 수 있으며 드물게는 구갈, 감각 이상, 손발저림, 경련, 의식저하 등의 부작용이 있을 수 있습니다. 이러한 부작용은 예측이 가능하며 대부분 자연 호전됩니다. 경련이나 의식저하 등으로 입원 치료를 하였던 경우도 아주 드물게 보고되고 있지만 대부분 보존적인 치료로 회복이 가능하였으며 이러한 장정결과 연관된 증상이 발생할 시에는 원인 교정을 위해 혈액검사를 시행하고 입원 등 치료를 위한 필요한 조치를 취하게 됩니다. 신독성은 일반적으로 5% 전후에서 발생하는 것으로 보고되고 있고 두 군 모두에서 유사할 것으로 예측합니다. 대부분 수액 공급 등 보존 치료로 호전됩니다.

② 장정결제를 복용한 후 대장내시경을 시행하였을 때 장정결이 불량하여 대장내시경 재 검사를 받을 수 있습니다. 불량한 정결상태를 보였던 경우를 분석해 보면 약제를 끝까지 다 복용하지 못하거나 대장내시경 시행 이전 섭취한 음식과 연관이 많아 대부분 수검자와 관련된 요인이 많습니다. 따라서 적합한 장정결 상태를 보이기 위해서는 장정결제 복용과 관련된 주의 사항을 잘 지키고 의료진의 지시대로 성실하게 정결제를 복용하는 것이 중요합니다.

③ 그 이외에 예측 가능한 부작용들은 대장내시경을 시행 받거나 대장폴립절제술을 시행 받을 시에 발생할 수 있는 시술 합병증으로서 출혈, 천공 등이 있습니다. 하지만, 이런 합병증들은 대장내시경 시술 자체에 의한 합병증이지 장정결제 복용에 의한 합병증은 아니기 때문에 본 연구와 관련된 합병증들이라고 하기 어렵습니다.

2) 부작용 보고 방법

임상시험 부작용 모니터

이 름: 김규원 (연구간호사)

주 소: 광주광역시 동구 필문대로 365 조선대병원 소화기내과

전 화: 062-220-3012

**7. 연구 참여에 따른 혜택**

이 시험에서 투여되는 모든 임상시험용의약품은 무상으로 제공되며, 1회 방문시마다 각 2만원씩 총 2회의

교통비가 지급됩니다.

**8. 환자의 권리와 비밀보장 (개인정보보호 및 개인정보 제공에 관한 사항)**

자료 수집 시 환자에 대한 정보는 최소화하여 직접적인 정보 유출은 없을 것으로 판단되며 환자 이름과 주민번호는 명시되지 않을 것이며 환자 등록번호는 암호화 할 것이며, 환자 정보가 유출되지 않도록 환자 정보 보호를 할 것입니다. 환자 정보에 대한 접근은 연구책임자 및 공동연구자로 제한할 것이며 환자 정보 file은 암호화하여 잠금 장치에 보관하고 접근이 제한된 컴퓨터에 저장, 접근 암호를 제한하고 책임연구자만 접근 가능하도록 할 것입니다. 모든 정보는 연구가 종료 후 3년간 보관 후 폐기될 것입니다.

**9. 연구 참여로 인한 피해발생시 조치사항과 피해보상 규정**

연구책임자는 본 임상연구 실시에 대하여 다음 사실을 확인해야 합니다.

- 본 연구 실시 중에 본 연구로 인해 이상반응 등 예기치 않은 사고 등이 발생하여 이에 대한 치료 또는 입원이 요구되거나 피험자 또는 보고자와 분쟁이 발생하는 경우에는 연구자가 그 비용을 부담합니다.

- 이러한 이상반응 처리는 다음 사항에 적합하여야 합니다.

첫째, 책임자 및 담당자가 본 임상연구계획서 내용을 충실히 이행하여야 하며

둘째, 본 시험 실시에 대한 책임자 및 담당자의 태만이나 의도적 또는 중대한 과실이 인정되지 말아야 하며

셋째, 발생한 이상반응에 대하여는 연구자에게 즉각적인 연락을 취하여 이에 대한 준비를 하도록 하여야 합니다.

- 다만, 부작용이 발생하였을 경우에도 임상시험과 무관하게 대장내시경 검사 자체에 의한 부작용으로 판단되었을 때에는 보상하지 않습니다.

**10. 연구 중단 사유:** 다음의 경우 연구책임자의 판단 하에 연구가 중단될 수 있습니다.
 * 효과불충분으로 책임자가 중지가 필요하다고 판단한 경우
 * 이상반응이 발현되어 책임자가 중지가 필요하다고 판단한 경우
 * 등록 후 피험자가 투여 및 검사를 거부하거나 동의를 철회하는 경우
 * 등록 후 대상자로 부적절하다고 판단한 경우
 * 등록 후 대상자의 사정상 검사 또는 연구의 실시가 불가능하다고 판단한 경우

**11. 연구 문의**

본 연구에 대해 질문이 있거나 연구 중간에 문제가 생길 시 다음 연구 담당자에게 언제든지 연락하십시오.

연구 책임자(성명): ______이 준_________ 전화번호: _____062)220-3012______

또는

**만일 어느 때라도 연구대상자로서 귀하의 권리에 대한 질문이 있다면 다음의 조선대학교병원 기관생명윤리위원회에 연락하십시오.**

**조선대학교병원 기관생명윤리위원회(IRB) 전화번호: 062-220-3268**

**끝까지 읽으셨다면 연구담당자(연구원)에게 충분히 질문하고 참여 여부를 결정해 주시기 바랍니다.**

**본 설명문은 동의서 사본과 함께 귀하에게 1부를 드립니다. “연구대상자용 설명문과 동의서(사본)”을 연구 참여 종료 시까지 보관하셔야 합니다.**
