# Supplementary material for: Comparison of optimal bowel cleansing effects of 1L polyethylene glycol with ascorbic acid versus sodium picosulfate with magnesium citrate: A randomized controlled study
Source: PLoS One. 2022 Dec 30;17(12):e0279631. doi: 10.1371/journal.pone.0279631 (PMC9803231; doi:10.1371/journal.pone.0279631)
Supplement: S2 File — (ZIP) [file pone.0279631.s003.zip › Study protcol, statement, consent form, consort check list/Statement for consent of clinical trial subjects(English).docx]

Statement for consent of clinical trial subjects

Tile: **Randomized clinical trial on efficacy and safety of 1L polyethylene glycol with ascorbic acid versus sodium picosulfate with magnesium citrate as preparation for colonoscopy**

This study is a study on the bowel cleansing effect, dosing compliance, and stability of a 1L ascorbic acid-containing polyethylene glycol (PEG) formulation and a magnesium citrate-containing picosolution formulation as a pretreatment laxative for colonoscopy. Since this clinical trial is a research study, there are experimental aspects that have not been verified. You should read the questionnaire and consent form carefully before deciding whether to participate in this study or not. It is important that you understand why this research is being done and what it does. ___Lee Jun___ Principal Investigator (Research Nurse Kim Kyu-Won or _Seong-Jung Kim), who conducts this study, will explain the study to you. This study will be conducted only for those who have voluntarily expressed their intention to participate. Please read the following carefully and express your intention to participate. If necessary, discuss it with your family and friends. If you do not have someone to discuss with, you can request an impartial witness to the Institutional Bioethics Committee of Chosun University Hospital. Your researcher will be happy to answer any of your questions about this study in detail. Your signature means that you have been told about the study and the risks and that you (or your legal representative) wish to participate in this study.

**1. Background and purpose of the study**

The incidence of colorectal cancer in Korea is 69.3 in men and 45.9 in women per 100,000 people, making it the third most common cancer after thyroid and gastric cancer. Since more than 80% of colorectal cancers progress to the adenoma-cancerous process, removal of adenomas through colonoscopy can significantly lower the incidence and mortality of colorectal cancer. However, even though colonoscopy was performed at appropriate intervals, it has been reported that about 6-8% of intermediate cancers occur. Insufficient bowel preparation can increase the detection rate of adenomas, lengthen the procedure time, cause cost and time loss, and ultimately become a major cause of middle cancer. An ideal bowel preparation should have high efficacy, ensure stability, and have high medication compliance. The conventional low-dose laxative reduces the capacity of the laxative by half compared to the existing 4L polyethylene glycol, but it has the disadvantage of requiring a large amount of water. Recently, 1L PEG plus ascorbic acid, an ultra-low-dose laxative, has been newly launched, which reduces the amount of laxative by half by increasing the content of ascorbic acid in existing polyethylene glycol. Compared to the existing 2L low-dose laxative (polyethylene glycol containing ascorbic acid), the 1L ascorbic acid-containing polyethylene glycol (PEG) formulation, which is an ultra-low-dose laxative, has improved the effect of bowel cleansing and the patient's re-taking preference, And similar results were confirmed in terms of side effects. However, to date, comparative studies between 1L ascorbic acid-containing polyethylene glycol (PEG) formulations and other low-dose laxatives are lacking. This study is a comparative analysis of ultra-low-dose laxatives (1L) and conventional low-dose laxatives. 1L 1L polyethylene glycol (PEG) formulation containing ascorbic acid (Cleanviewal powder, Taejoon Pharm. Co, Seoul Korea; and magnesium citrate-containing picosolution formulations (Picosolution, Pambio Co, Seoul Korea, Picosolution, Pambio Pharm) to evaluate the effect, stability, compliance, preference and satisfaction of bowel preparation after administration.

**2. Number of study participants**

In this study, 250 people from 4 university hospitals, including this institution, will participate in the study.

**3. Research Methods and Prediction Results (Effect)**

Although this study is prospective, it does not take any form other than the actual examination method for the study, and it does not affect your current or future examination and treatment in any way. You will prepare your bowels using either a 1L polyethylene glycol (PEG) formulation containing ascorbic acid or a picosolution formulation containing magnesium citrate. Colonoscopy and treatment are the same as general examinations, but medical records including endoscopy performed by you at our hospital for diagnosis and treatment are read by us, and some of them may be cited in research papers. However, even if there is information cited in the paper for research purposes, the patient's personal information will be thoroughly protected.

**4. Duration of study participation**

The total study period of this exam is expected to be 12 months (1 year from the date of approval).

**5. Dropout during study participation**

You may quit at any time even after participating in the study. If you wish to stop participating in the study, please notify the researcher or principal investigator immediately.

**6. Possible side effects (risk factors) due to study participation**

1) Evaluation criteria and methods for side effects

In this clinical trial, adverse events refer to any unpredictable medical problems that may occur during the clinical trial. If side effects that require hospitalization, such as convulsions, severe dehydration, and loss of consciousness, occur after taking the intestinal tablet, the research team in charge must immediately report to the IRB and the clinical trial headquarters in hand. After review by the co-researchers, the exclusion of subjects, exclusion of research institutes, or termination of the study is denied. In a recent multicenter study conducted in a foreign country, we analyzed the difference between the low-dose laxative group and the PEG laxative group, including elderly patients up to 80 years of age, and no statistical difference was observed. Therefore, the predicted side effects/complications are also expected to be within the range of the existing side effects/complications side effects of the two groups of enteric tablets that are being implemented in clinical practice. The use of bowel preparation before the procedure in this study is an essential pretreatment that is currently approved for colonoscopy in Korea. As both the 1L ascorbic acid-containing polyethylene glycol (PEG) formulation or the magnesium citrate-containing picosolution formulation are bowel preparations already used in clinical settings, there is no possibility that additional risks will be added by the study.

① Vomiting, abdominal distension, abdominal pain, dizziness, etc. may occur while taking or after taking all of the bowel preparation. These side effects are predictable and usually get better on their own. Cases of hospitalization due to convulsions or loss of consciousness have been reported very rarely, but most of them can be recovered with conservative treatment. action will be taken. Nephrotoxicity is generally reported to occur in around 5% and is predicted to be similar in both groups. Most improve with conservative treatment, such as fluid supply.

② When a colonoscopy is performed after taking a bowel preparation, the colonoscopy may be re-examined due to poor bowel preparation. If we analyze the cases of poor cleanliness, most of the factors are related to the examinee as it is related to the inability to take the drug to the end or the food eaten before the colonoscopy. Therefore, in order to show proper bowel preparation, it is important to observe the precautions related to taking the bowel preparation and to take the preparation faithfully as directed by the medical staff.

③ Other predictable side effects include bleeding and perforation, which can occur during colonoscopy or polypectomy. However, it is difficult to call these complications related to this study because these complications are due to the colonoscopy procedure itself and not the complications caused by the use of bowel prep medication.

2) How to report side effects

Clinical Trial Adverse Event Monitor

Name: Kyuwon Kim (Research Nurse)

Address: Department of Gastroenterology, Chosun University Hospital, 365 Pilmun-daero, Dong-gu, Gwangju

Tel: 062-220-3012

**7. Benefits of Participating in Research**

All investigational drugs administered in this trial are provided free of charge, and each visit is worth 20,000 won for a total of 2 times.

Transportation is paid.

**8. Patient Rights and Confidentiality Protection (Personal Information Protection and Provision of Personal Information)**

When collecting data, it is determined that there will be no direct information leakage by minimizing patient information. Access to patient information will be restricted to the principal investigator and collaborator, and the patient information file will be encrypted and stored in a lock, stored on a computer with restricted access, and access password will be restricted and accessible only to the principal researcher. All information will be stored for 3 years after the end of the study and then destroyed.

**9. Measures to be taken and rules for compensation for damage caused by research participation**

The principal investigator must confirm the following facts regarding the conduct of this clinical study.

- In the event that an unexpected accident such as an adverse reaction occurs due to this study during the course of this study, treatment or hospitalization is required, or if a dispute arises with the subject or reporter, the researcher shall bear the cost.

- Such adverse reaction treatment must comply with the following items.

First, the person in charge and the person in charge must faithfully implement the contents of this clinical research plan.

Second, negligence or intentional or serious negligence of the person in charge and person in charge of conducting this test should not be recognized.

Third, for any adverse reactions that have occurred, the researcher should be immediately contacted to prepare for it.

- However, even if a side effect occurs, no compensation will be provided if it is judged to be a side effect by the colonoscopy itself regardless of the clinical trial.

**10. Reasons for study discontinuation**:
 The study may be discontinued at the discretion of the principal investigator in the following cases. * When the person in charge decides that it is necessary to stop because of insufficient effect * When the person in charge decides that it is necessary to stop because an adverse reaction occurs In case * After registration, if it is judged that it is impossible to conduct examination or research due to the circumstances of the subject

**11. Research Inquiries**

If you have any questions about this study or if you have any problems during the study, please feel free to contact the following research staff:
