# Supplementary material for: Comparison of optimal bowel cleansing effects of 1L polyethylene glycol with ascorbic acid versus sodium picosulfate with magnesium citrate: A randomized controlled study
Source: PLoS One. 2022 Dec 30;17(12):e0279631. doi: 10.1371/journal.pone.0279631 (PMC9803231; doi:10.1371/journal.pone.0279631)
Supplement: Attachment — Submitted filename: Response to Reviewers.docx [file pone.0279631.s004.docx]

October 9, 2022

Manuscript Number: PONE-D-22-01108

Manuscript Title: **Comparison of Optimal Bowel Cleansing Effects of 1L Polyethylene Glycol with Ascorbic Acid versus Sodium Picosulfate with Magnesium Citrate: A Randomized Controlled Study**

On behalf of all the authors, I would like to express our sincere gratitude for your thorough consideration and evaluation of our manuscript titled “Comparison of Optimal Bowel Cleansing Effects of 1L Polyethylene Glycol with Ascorbic Acid versus Sodium Picosulfate with Magnesium Citrate: A Randomized Controlled Study”. The appropriate and helpful comments made by Reviewers have allowed us to address the important issues in this paper. After reviewing the manuscript to the best of our ability to the reviewer's comment, we revised the manuscript. The revised manuscript is the result of our efforts and sincerely hope that our revision will be considered favorably during further review. We are aware of the importance of the Reviewers’ assessment of the scientific and clinical merit of the manuscript. The changes in the revised manuscript have been highlighted (in yellow text). Specific responses to the Editor’s and Reviewers’ comments are included below.

**1.Reviewer #1:** This paper represents a well-written scientific work. The English language is correct and appropriate. Various other investigations were published in this yield, so the subject can stimulate scientific discussion poorly. For this reason, I 'm not fully convinced to support the progression of publication process

**Author’s response:**

Thank you for your valuable comment. As the reviewer pointed out, various studies on 1L PEG formulations are being actively conducted. However, most studies compared 2L PEG formulations, and very few studies compared non-PEG low-dose formulations. In particular, both studies comparing sodium picosulfate with magnesium citrate have clear limitations and are unreliable. Therefore, we believe that our well-designed study will be of great value and will be of great help to clinicians in choosing bowel cleansing agents

To emphasize the importance of our study, the introduction has been revised and a table 1 has been inserted.

**Please see the line 68-75. Table 1.**

**2. Reviewer #2:** A two-arm randomized controlled study was conducted which aimed to compare the effectiveness of PEG to PICO for colon cleansing. The rates of overall bowel cleansing were similar in the two arms

**2-1) Major revision**

- **Reviewer’s Comment #2-1-1**

Include a comprehensive statistical analysis section which lists and describes all the statistical testing methods used to generate the results

**Author’s response:**

Thank you for your comment. As your comments, we have revised the "statistical analysis" section.

Please see the line 144-160

- **Reviewer’s Comment #2-1-2**

: State the statistical testing method which archives 80% power. Perhaps it was a chi-square test. Thus the trial was designed as a superiority trial, instead of a non-inferiority trial as noted in the abstract. Rectify these differences. Either the trial is a superiority study or a non-inferiority study, not both

**Author’s response:**

Thank you for your valuable comment

This study design corresponds to a study in which the standard treatment was established rather than the superiority of the new treatment. Therefore, a non-inferiority test was performed to determine whether the new formulation (1L PEG with ascorbic acid) had a similar effect to the standard treatment(Sodium picosulfate with magnesium citrate).

80% power was designated with reference to the existing non-inferiority test study that identified the therapeutic effect (efficacy and safety) of 1L PEG. Therefore, we added two reference

Please see the line 148 (reference 13,18)

2-2) Minor revision

- **Reviewer’s Comment #2-2-1**

The standard statistical term for average is mean.

**Author’s response:**

Thank you for your valuable comment. As your comments, we revised the text.

Please see the line 178 (average -> mean)

- **Reviewer’s Comment #2-2-2**

Line 167: Provide standard deviations corresponding to the mean ages in the two arms.

**Author’s response:**

Thank you for your valuable comment. As your comments, we added to the text

Please see the line 179. (53.8±12.4 years and 53.7±10.6 years)

- **Reviewer’s Comment #2-2-3**

3- Line 181: More clearly describe the LCL. It appears to be the lower confidence limit for the difference in bowel cleansing rates.

**Author’s response:**

Since we performed a non-inferiority test, we presented a lower confidence limit.

- **Reviewer’s Comment #2-2-3**

Explain all acronyms and abbreviations at first mention.

**Author’s response:**

Thank you for your valuable comment. As your comments, we revised the text.

Line 38/41. LCL -> lower confidence limit

Line 113. h-> hour(h)

Line 167. COVID19-> the coronavirus disease 2019 (COVID-19)

Line 255 FDA-> Food and Drug Administration (FDA)

In conclusion, we have endeavored to thoroughly answer and address all the issues raised by the Reviewers and appreciate their comments which have greatly strengthened the quality of our manuscript. The authors welcome any further constructive suggestions that may improve the quality of the manuscript.

Sincerely,

Jun Lee, MD, PhD

Professor

Department of Internal Medicine, Chosun University Hospital

365, Pilmundaero, Gwangju 61453, Korea

Office: +82-62-220-3016, Fax: +82-62-224-5494

Email: [leejun@chosun.ac.kr](mailto:leejun@chosun.ac.kr)
